# Social Health and Psychological Safety of Students Involved in Online Education during the COVID-19 Pandemic

**DOI:** 10.3390/ijerph192113928

**Published:** 2022-10-26

**Authors:** Elena Korneeva, Wadim Strielkowski, Raisa Krayneva, Anna Sherstobitova

**Affiliations:** 1 Department of Mass Communications and Media Business, Financial University under the Government of the Russian Federation, Leningradsky Prospekt, 49, 125993 Moscow, Russia; 2 Institute of Finance, Economics and Management, Togliatti State University, Belorusskaya str. 14, 445020 Togliatti, Russia; 3 Department of Trade and Finance, Faculty of Economics and Management, Czech University of Life Sciences Prague, Kamýcká 129, 16500 Prague, Czech Republic

**Keywords:** university students, public health, online learning, psychological safety, sustainable education, COVID-19 pandemic

## Abstract

Our paper focuses on the issues of social health and psychological safety of university students involved in digital sustainable education during the COVID-19 pandemic. Currently, modern education is becoming inclusive due to the advancements in information and communication technologies (ICT), and it is important not only to stress the relevance of sustainable development and the use of digital technologies, but also their impact on students at schools and universities worldwide. Digital literacy is a newly emerging feature that results from the attitude of team members in the field of digital technologies. This paper explores the impacts of the COVID-19 pandemic on students’ learning and well-being and outlines the potential considerations for educational systems as they support students through the recovery period and beyond. Our study is based on the results of our own survey that was administered using a snowball and convenient sample of 1524 respondents (aged 19–26 years; 56.2% females and 43.8% males) from the Czech Republic (N = 804) and Russia (N = 720). We employed the ANOVA and Dirichlet Process mixtures of Generalized Linear Models (DP-GLM) in order to explain the causes of stress and anxiety after grouping variables represented by gender and the study specializations. Our results demonstrate that more than 87% of the students in the sample expressed a medium to high vulnerability to stress, while 58% of the respondents were affected by severe anxiety during their online education engagement. The most important factors that emerged as significant were the fear of getting infected and social distancing, while the best strategy to cope with the stress was self-control. These results allow us to provide practical recommendations for effectively coping with and controlling stress and anxiety among students in the post-pandemic era. In addition, our findings might contribute considerably to the study of the overall long-term effect of the COVID-19 pandemic on the university students, in general, and the use of digital technologies in higher education, as well as on the public health.

## 1. Introduction

The COVID-19 pandemic led to a digital revolution in higher education, with such novel features as online remote lectures, digital open books, online exams, and interaction in virtual environments [1,2]. The pandemic created challenges and caused disruptions throughout the higher education sector: college campuses closed, and face-to-face instruction and assessment were moved online [3]. The pandemic also highlighted the adaptability of both the scholars and the students within higher education, who were set to continue to embrace online learning and assessment [4]. Furthermore, the pandemic opened the door to the new technologies that would endure in the classroom after the disaster subsided [5]. COVID-19 highlighted that the higher educational institutions (HEIs) were crucial as the providers of education, as well as being spaces for responding to socio-emotional needs and for supporting the well-being of vulnerable students [6,7]. Due to the lockdowns and the necessity to conduct distance learning, many institutions started using educational technology driven by artificial intelligence (AI) that predicts the navigational behavior of learners, allows for the real-time identification of pedagogically valuable behaviors, and acts as an online personalized tutor that can assess students’ strengths and weaknesses and deliver individual instructions [8,9].

However, the pandemic also brought with it an increase in the anxiety and the stress levels of students in many countries around the word [10,11,12]. The lockdowns, which led to loneliness caused by the need to isolate oneself, either in quarantine when the COVID-19 virus was detected or suspected, or as a preventive measure to stay healthy, had some significant impacts on students’ mental health and feelings of well-being. In turn, this psychological discomfort often resulted in problems with learning and absorbing new information and skills, as well as in the deterioration of the usefulness of the digitalized online learning content [13,14].

In the years leading up to the pandemic, many HEI systems faced challenges with respect to providing high-quality educational opportunities to the majority of their students (including the comfort of lecture rooms, good infrastructure, food opportunities, or accommodation facilities for the students and faculty). However, COVID-19 is the first global pandemic to hit the HEIs in recent times, and the experience has definitely been challenging for academics as well as students [15,16]. Many students suffered in quarantine and were worried about the effects of the crisis on their educational outcomes because the universities were closed during the COVID-19 pandemic. One of the COVID-19-related stressors, anxiety, which is related to the educational impact, often became positively associated with the anxiety symptoms amongst the students during the pandemic [17]. At the same time, female students frequently experienced higher levels of stress and depression compared to male students due to the fear of losing their educational gains due to the shutdowns, and also because of the fear that COVID-19 would have negative effects on their families’ or relatives’ well-being [18]. The pandemic and the resulting sudden changes in their everyday lives and their ways of studying has had significant impacts on college and university students, particularly affecting their mental health. 

Additionally, beyond the academic impacts, the pandemic had wider societal and emotional impacts for students around the world: mental-health issues were increasing, reports of violence against children emerged more often, obesity was increasing as was teenage pregnancy, and higher levels of chronic absenteeism and higher numbers of school dropouts resulted [19,20,21]. 

In addition, after months of mandatory online instruction, the majority of the students expected that they would have lower educational outcomes from the online instruction than from the on-campus instruction prior to the pandemic [22]. Following the lockdowns, the highest priority was given to online education in order to continue academic activities and prevent high school students from dropping out. The idea of using digital technologies for teaching students from home was introduced for continuing with education and overcoming the psychological pressures and anxieties that occurred when the full shutdown transpired. However, as the university and college professors were pushed headlong into providing education for their students exclusively via a digital interface, many of them found this was an uncommon, disorienting, or even an undesirable experience. It is possible that many students had just the same experience, which was also exacerbated by the lack of the study discipline that is crucial in the first years of university education [23,24]. 

For many students, their concerns about health and safety made studying harder, their academic enthusiasm faded, and their dim hopes for future employment eroded program completion [25]. Although the students experienced the digital revolution overnight, it was hard to understand much about their experiences after the universities closed down their campus activities; most countries worldwide closed their educational institutions temporarily to try and curb the spread of the coronavirus and to decrease infections. The population’s psychological distress was highest in the periods of increased COVID-19 deaths and the pandemic containment measures [26].

The novelty of our study is that while a number of studies have addressed depression, anxiety, and stress among the college students in such countries as China, Germany, Poland, Romania, or the Ukraine, research in such countries as the Czech Republic and Russia was quite rare. Our paper attempts to address this by using a unique sample of our own data from both the Czech Republic and Russia that represents these two distinct countries that are both quite similar (a socialistic Soviet past and the thorny path of economic and political transition), but also quite different in political and economic terms, especially at the moment.

In this paper, we studied the causes of stress and anxiety for university students from the Czech Republic and Russia subjected to sustainable digital education during the COVID-19 lockdowns. Specifically, we focused on the grouping variables represented by gender and the study specialization of the students during their online education. This paper is organized as follows: Section 2 offers a review of the relevant academic literature related to the social health and psychological safety of students in the digital post-COVID era. Section 3 presents the basic information about our survey and data. Section 4 outlines the main variables, sets up the empirical model, reports the main results, and provides the comprehensive discussion of these results. Finally, Section 5 concludes the paper by listing the study’s main outcomes and implications, as well as the pathways for further research.

## 2. Literature Review: COVID-19 and the Mental Health and Psychological Safety of Students 

The COVID-19 pandemic had a major impact on public health all around the world [27,28,29]. Moreover, it also caused many educational and social impacts such as university campus closures and the implementation of online learning and social distancing at HEIs all around the world [30,31,32]. 

Several studies from across the globe measured depression, anxiety, and stress levels among university students [33,34,35]. However, very few of these studies measured depression at the time of home isolation and the COVID-19 outbreak. One notable contribution is that female college students scored higher on measures of depression, stress, and anxiety compared to male students and this was also true prior to the outbreak [36,37].

In some European countries, such as the United Kingdom, France, and Greece, increases in anxiety and depression were observed during the pandemic, and especially during the lockdown periods, among university students [38]. One survey conducted in late May–early June 2020 found that 85 percent of undergraduate students experienced increased anxiety and stress levels during COVID-19, yet only 21 percent of respondents sought out licensed counselors or professionals [39]. According to a Healthy Minds Networks survey (2020), which collected data from 14 university campuses throughout the U.S. from March–May 2020, the proportion of students experiencing depression increased by 5.2 percent from the year prior [39]. Nevertheless, there are few studies that focused on college students and their mental health during COVID-19, and most national surveys conducted across the United States did not use clinically validated tools for measuring the students’ mental health [40]. Given that some studies show that college students are particularly at risk of negative mental health outcomes, these concerns are likely to continue and in fact, could potentially be intensified, due to the pandemic. In addition, it should be noted that although empirical studies conducted in many HEIs worldwide have clearly demonstrated that undergraduate students were experiencing severe psychological distress during COVID-19, many of them were relatively small in sample size and seldom investigated whether specific groups were more vulnerable than others during the pandemic [41,42]. According to some studies, higher levels of anxiety, depression, and stress typically influence the individuals with higher levels of education, and this correlation makes college students a particularly pertinent sample to assess the effects of the pandemic on anxiety levels [43]. In addition, other studies confirm that COVID-19 and its associated disruptions resulted in a substantial increase in stress, anxiety, depression, and suicidality among university students. In families where either a high school or a college student experienced educational disruption due to COVID-19, other family members reported increased stress as a result of that disruption [44,45,46,47]. 

Furthermore, consistent with the prior studies of college students’ emotional well-being during COVID-19, a substantial share of students reported difficulties in dealing with the disruptions associated with the pandemic and the increased levels of stress. This compounded the preexisting mental health crises at colleges; COVID-19 may significantly complicate a schools’ efforts to support students experiencing increased mental distress and economic hardship affecting their enrollment status [48,49,50]. 

While it is probably too soon to know if the disruptions caused by COVID-19 will affect higher education enrollment, colleges should be proactive in responding to the challenges posed by the students’ anxieties and isolation, especially in the underserved populations. With the exception of the highest-stress categories, the reported rates of student depression across multiple recent studies were quite high, suggesting a possible increase in depression symptoms related to the pandemic among undergraduate students [51,52]. 

More broadly, many academic studies revealed that adults reported decreased physical activity and an increased food intake during the COVID-19 pandemic lockdowns, relative to the pre-pandemic periods, as well as increased binge drinking, and on average, that was also identified among a smaller proportion of the undergraduate respondents. Although most students expressed concerns about their academic performance, it is noteworthy that nearly half of them reported lower levels of stress related to academic pressures and classroom workloads after the start of the COVID-19 pandemic [53,54,55]. 

For some students and families, the COVID-19 pandemic offered a sense of security and reassurance [56,57]. For the students, home has become a safe learning environment where they can feel productive. Moreover, while universities are places where young people can socialize and make friends, not all social interactions are positive [58]. The incidence of mental health problems among students in higher education is similar to the incidence in the general population. The stress of studying can lead to emotional stress for students, whether they are in a traditional classroom or a distance school class [59,60]. Being a teenager or a young adult is challenging enough, but the extra stress on students can exacerbate normal fears and stressors. This is why the development of mental health and well-being resources for faculty and students is crucial for universities and university administrations. Investing in the creation of campus policies that recognize that mental illness and other illnesses can occur, and in providing training to faculty on how to address these issues, can increase the online student engagement and success. 

Our study was designed to evaluate the prevalence and predictors of depression, anxiety, and stress in college students in the Czech Republic and Russia during the time of the COVID-19 home isolation and online learning. Consistent with this, the psychological distress levels measured in our study were associated with the students’ concerns regarding their academic activities, both with respect to delays regarding degree completion and with their feelings of loneliness and isolation because of the physical distance from parents and friends as a result of the effects of COVID-19 and the containment measures applied.

## 3. Survey and Data

The data obtained for testing our empirical model were collected by the research team in the Czech Republic and Russia. A total of 1524 respondents completed a questionnaire voluntarily and anonymously using a prepared online Google Docs form in a link provided in a personalized e-mail message. All participants were Czech and Russian native speakers residing in their respective countries throughout the pandemic. The method of sampling contained the elements of the snowball technique and opportunity sampling. Due to the methodology of the sample construction described above, our sample does not assume to be representative as such. A total of 6 participants were excluded from further analysis due to incomplete answers. The resulting sample included 1518 respondents (aged 19–26 years; 56.2% females and 43.8% males).

All of our questionnaires were filled out via the Google Docs form available on the Internet during the period from November 2021 to February 2022. The reason behind this approach is that it had the ability to reach the Internet-based population, which otherwise would not be reachable [61,62], and enables communication with people who are reluctant to meet face-to-face [63]. 

Our questionnaire survey consisted of 20 questions used to measure stress scores: among them, one question was intended to identify the most important stressor during the pandemic (fear of virus, fear of vaccine, social distancing, mask wearing, online lectures, and overwork for employees); one question was used for identifying the most efficient strategy to cope with stress during the epidemic (self-control, family support, colleagues, professors and friends, and spiritual support (belief in God, meditation, etc.)); one question with five sub-categories was used to measure the anxiety score (the degree in which the following aspects were affected: working capacity, work in the household (such as cleaning, cooking, or shopping), recreational social activities, individual recreational activities, and maintaining close ties with others); one question related to the living environment (living alone, with parents, with other relatives, renting or living in the student guesthouse); one question related to the specialization of studies (social sciences, economics, business and finance, information technologies, engineering, and medicine); as well as several socio-demographic questions (age, gender, etc.). The responses related to the measures of anxiety were on a scale from 0 to 8 and the responses related to vulnerability to stress were on a scale from 1 (always) to 5 (never).

All participants were informed that the data they provided was confidential and used for research purposes only, and would not be transferred to third parties. All subjects gave their informed consent for inclusion before they participated in the study. The study was conducted in accordance with the Declaration of Helsinki, and the protocol was approved by the Ethics Committee of the Czech University of Life Sciences Prague, project No. VEV0310/2021. All the participants participated voluntarily and anonymously in the present study. All the participants signed the informed consent for participation in the study.

Table 1 presents the distribution of students according to various characteristics split by the two countries (Czech Republic and Russia). 

More than 80% of the students in the sample proved to have a medium to high vulnerability to stress, while 64% of the individuals presented severe anxiety. Fear of the virus and social distancing were among the most-mentioned factors of stress for the students: students in both countries mentioned these factors. More than half of the students in the sample considered self-control as the most important strategy for coping with stress during the COVID-19 pandemic. About 40% of the students in both countries were 20 years old with about 80% of students in both countries (88% in the Czech Republic and 80% in Russia) were no older than 21 years. Even though it might seem interesting to present the data by the age subgroups, we did not do it for the reason mentioned above. Nevertheless, our results suggest that the impact of the pandemic was less among the first-year students than for those in their final year, as the first-year students appeared to be less vulnerable compared to the final-year students (more than 70% of the students aged 23 and older in both countries marked such factors as social distancing and online lectures as their major factors of stress). Surely, this issue represents a worldwide trend when the transition from the ‘good old habits’ of in-class teaching to the online version might be especially painful for those students who are about to complete their degrees, as some other related studies suggest [64,65].

The students’ gender distribution was 56.2% females and 43.8% males. The majority of students studied economics, business, and finance (35% in the Czech Republic and 28% in Russia), which was followed by the information technologies (28% in the Czech Republic and 32% in Russia), and the social sciences. The noticeable difference was that the majority of the students in the Czech Republic were renting their apartments (60%), while the majority of the students in Russia were staying in the student dorms (37%). Otherwise, the differences in the students’ perceptions and attitudes were minimal, which gives us grounds to analyze the merged sample of students for better validity of results.

## 4. Results and Discussions 

In this paper, we used the ANOVA and Dirichlet Process mixtures of Generalized Linear Models (DP-GLM) for explaining the causes of stress and anxiety after grouping variables represented by gender and the study specialization. Our analysis was based on the methodology originally used by Karabatsos and Walker [66]. Let us consider the variables $X={\left(\left(1,x_{i}^{T}\right)\right)}_{nx\left(p+1\right)}$ and $y={\left(y_{1},\ldots ,y_{n}\right)}^{T}$. In this case, *i* = 1, …, *n* is used as index for students in the sample. For one constant (1) and *p* covariates, $x={\left(1,x_{1},\ldots ,x_{p}\right)}^{T}$, the regression parameters are denoted by $\beta ={\left(\beta_{0},\beta_{1},\ldots ,\beta_{p}\right)}^{T}$, where $\beta_{0}$ is the intercept and $\beta_{1},\ldots ,\beta_{p}$ represent the slopes associated with covariates and $\sigma^{2}$ is the error’s ($\varepsilon_{i}$) variance. The general form of a non-parametric model is given by the following Equation (1):

(1) $$f\left(y|x;\;\vartheta \right)=\int f\left(y|x,\tau ,\theta \right)dG_{x}\left(\theta \right)=\overset{\infty }{\underset{j=1}{\sum }}f(y|x,\tau ,\theta_{j}\left(x\right)\omega_{j}\left(x\right)$$

where {f(.|x,τ,θ)}:(θ,τ)}∈Θ represents kernel densities; $\omega_{j}\left(x\right)$ is mixing weights of unitary sum for any x∈ϰ; $\delta_{\theta \left(x\right)}\left(.\right)$ probability measure which degenerates at θ(x); τ- other coefficients that do not belong to the mixture; ${\left\{\omega_{j}\left(x\right)\right\}}_{j},{\left\{\theta_{j}\left(x\right)\right\}}_{j}$ infinite collections of processes indexed after ϰ. The prior distribution corresponding to coefficients of the Bayesian density regression model can be expressed as follows (2):

(2) $$\vartheta =\left(\tau ,{\left(\omega_{j}\left(x\right),\theta_{j}\left(x\right)\right)}_{j}\right),\;\;\;x\in \;\varkappa$$

The Dependent Dirichlet Process (DDP) is employed by many Bayesian density regressions. The DDP prior is represented as $G_{x}\sim DDP\left(\alpha ,G_{0x}\right)$.

Table 2 above presents the results of the correlation between stress scores, anxiety scores, factors of stress, and strategies for coping with stress and various economic and demographic variables (age, environment, gender, and specialization) which are analyzed using the chi-square test. We conducted the estimations of students from both countries (Czech Republic and Russia) in our sample together (in this model and in the subsequent estimations and presentations of results) due to the fact that the results for both countries were quite similar, which gave us the reason to merge them and analyze them together. It appears that presenting the data for each country and analyzing them separately would not have led to the statement of the other conclusions.

The results in Table 2 suggest that factors of stress during the pandemic were associated with the students’ gender and age at the 5% significance level. A significant association was observed between strategies for coping with stress and stress and anxiety scores, specialization, age, gender, and environment. Stress and anxiety scores were strongly correlated, while the level of stress was associated with the specialization, age, and living conditions. 

Moreover, 31.1% of the students of 19 years old considered online lectures as the strongest factor of stress, while 30.8% of the people more than 22 years old claimed that social distancing was the most stressful situation in the pandemic. Social distancing was the most important issue for 29.3% of the females, and 31% of the males were more afraid of the possibility of contracting the COVID-19 virus. Furthermore, 58.1% of the students were living alone, with 60.1% of those living by rent; 75.8% of the students were living with parents, with 72% of those living with other relatives; and 51.9% of students who were in a guesthouse considered that self-control was the best strategy for coping with stress during the pandemic. More than half of the students in each category of age, in each category of stress, and in each category of anxiety, that is 68.3% of the females and 58.8% of the males, and more than half of the students from humanities, business, economics and finance, and information technologies indicated that self-control was the most efficient strategy for coping with stress. Additionally, it transpires that students with a high and an extreme vulnerability to stress also present with high anxiety. For example, 74.9% of the students with a high vulnerability to stress also have high anxiety, while 64.4% of those with an extreme level of stress present with high anxiety.

Before the Bayesian analysis, the indicators were normalized. Table 3 and Table 4 report the marginal posterior distribution for the intraclass correlation coefficient denoted by the intraclass correlation coefficient (ICC), which indicates the proportion of the variation in the scores for stress/anxiety that are caused by the between-groups heterogeneity. The independent variables are stress and anxiety, and the dependent variables include the environment, age, and the specialization.

On average, 76.3% of the variation in stress scores is due to the heterogeneity between males and females, while 82.6% of the variation in anxiety scores is also caused by this type of heterogeneity. Table 3 also presents the marginal posterior distributions of the reliabilities of the estimates of the random intercepts and random slope parameters over the groups.

In addition, 78.6% of the variation in the score for stress is subjected to heterogeneity between the study specializations, while 73.2% of the variation in the anxiety scores is caused by this heterogeneity. If the entire sample is analyzed, age, specialization, and environment are significant causes for stress, while none of the factors are causes for anxiety (see Table 4 that follows). 

Finally, the results of the group analysis revealed that labor status, environment, and age are associated with stress and anxiety (even though they might not be the causes), while gender is also a factor that might lead to anxiety. Therefore, it becomes clear that university students represent quite a vulnerable group that might exhibit various health and mental health-related problems that require immediate attention and support when something massive and unexpected, such as the COVID-19 pandemic, strikes and the usual ways of studying at HEIs and residing in dorms or any other student accommodation are distorted. The introduction of the online education surely presented a solution of how to maintain the operation of the HEIs during the COVID-19 pandemic, but it also caused adverse effects such as the feelings of loneliness and discomfort, as well as physical detachment from friends and colleagues. 

## 5. Conclusions 

Overall, the COVID-19 pandemic has introduced a substantial array of stresses to many students’ lives threatening their mental health and well-being. It becomes clear that efficient and inclusive access to specialized services and psychological help for vulnerable students might be needed. 

Our results from the online questionnaire survey administered in the Czech Republic and Russia between November 2021 and February 2022 revealed that more than 87% of the students in our sample yielded medium to high vulnerability to stress, while 58% of the students were affected by severe anxiety during the online education caused by the COVID-19 pandemic and lockdowns. The most important factors contributing to stress and anxiety were the fear of being infected and social distancing, while the best strategy to cope with the stress appeared to be self-control. This finding leads to the conclusion that sports and relaxation need to be promoted as a good basis for building and sustaining effective self-control.

Hence, a broader approach to well-being and staying mentally healthy (the one that will constitute the “new normal” after the COVID-19 pandemic) needs to consider the socio-economic impacts of the pandemic on families and wider communities, and the implications for higher education. In addition to the need to offer additional services for vulnerable students, the COVID-19 pandemic may be an opportunity to build a well-being culture at HEIs. Adopting a holistic approach to education should incorporate the students’ learning, social, and emotional needs, and require that governments work collaboratively with other relevant agencies, such as healthcare and community organizations, social service agencies, and other supportive services, to meet the complex needs of the more vulnerable students during and following the coronavirus crisis. The framework for supporting students, families, and educators in their return to school during and following the COVID-19 pandemic, with priority given to students’ health and safety, social and emotional needs, as well as other supports, and behavioral and academic growth needs to be created and sustained.

In the mobilization of addressing the effects of the COVID-19 pandemic on students’ learning and well-being, countries may also need to revisit their education systems: questioning what has worked well and what might need rethinking in the light of the last two years. A new education policy needs to be set in place to prepare the schooling systems for managing pandemics like this one in a more effective way in the future, with the aim of avoiding long-term disruptions, while moving toward building a stronger public education system. The lessons learned from the institutions and students’ experiences with remote teaching and learning during the COVID-19 pandemic would inform students’ expectations for future learning, teaching, and assessments, underscoring the need for universities to focus on their own unique competitive advantage. This new policy might take some time to implement, but the foundation stone has already been laid as the HEIs and students were able to experience the novel forms of learning and teaching and to assess their advantages and disadvantages in all spheres including public health.

We have to acknowledge the limitations of our study stemming from the non-random sample of students and the self-reporting nature of the survey administered to our respondents (not to mention that all the respondents originated from just two countries and the samples was quite modest in size). However, we still consider our results to be representative in terms of reporting interesting insights into the vulnerability to the stress and anxiety of Czech and Russian students. Some future research might attempt to obtain a more representative and larger sample of students from the countries involved in our study. Another useful pathway for further research would be to conduct a cross-country study involving the samples of students from many countries. In addition, given the fact that the majority of our respondents reported the fear of getting infected as their main concern, it would be interesting to study the impact of social networks and Internet content on spreading news and information on the COVID-19 virus. Quite often, students tend to read the posts or follow the news while following their online lectures and since a considerable proportion of the Internet content in 2020–2021 was related to the new coronavirus and its effects, it might be that this exposure further elevated the students’ fears and the levels of stress. 

Novel 21st century educational models, such as online education that is based on new technological advances, have become a powerful platform for transmitting valuable knowledge and sharing new ideas and insights. There is a variety of educational methods for meeting the different educational needs of today’s societies. The age of globalization has led to the rapid creation and dissemination of knowledge. Rapid technological developments have revolutionized the current educational model, which can also become a solution for how to foster higher education in developing countries that cannot afford modern university facilities and top-notch professors on their payrolls, but can easily arrange online lectures and tuition for a smaller margin of the price. Access to education is low in many developing countries, and inequalities persist in highly stratified developed countries (for example, the United Kingdom). The large influx of skilled workers from developing countries to developed countries (e.g., United States, Canada, the United Kingdom, or Australia) is expanding the gap between the development potential of developed countries and the development potential of developing countries. Online education can help to narrow this gap by improving the quality of education in the developing countries via e-learning and online lectures by the prominent specialists and professors based in developed countries, and transferring the necessary knowledge and skills. The COVID-19 pandemic demonstrated the advantages of online education and learning and showed that the red tape associated with their implementation can be removed literally overnight. However, shifting to this mode of learning and education can nevertheless bring many problems and issues related to the anxiety and mental health of the students, as the experience from the COVID-19 pandemic-induced online learning has demonstrated. 

The relevant stakeholders and policymakers need to take precautions for the future in case something similar to COVID-19 strikes again. This includes providing wellness, health, and medical-related support services that are responsive to student and staff needs in times of any hypothetical pandemic, where the physical use of social, recreational, sports, and cultural facilities are affected. It is essential to foster well-being in the university community through integrating health care into university cultures, structures, and processes, and by establishing healthier work, learning, and living environments for students and staff, especially during the time of a pandemic, where social distancing should not mean social isolation.

Online learning communities in higher education have developed as a result of the COVID-19 pandemic, reflecting both an increased and more diverse student population, and a need for teachers to upgrade their instructional skills, practices, and strategies in order to substitute virtual classes for traditional ones. Parents, faculty, and educational institutions have attempted to not only promote learning, but to offer social and moral support, and ensure interactions still occur even while schools are closed. Universities need to learn how to provide a safe, supportive learning environment for students, which supports social and emotional development, provides access to vital services, and enhances life outcomes. Mental health supports that encompass social–emotional learning, resilience, and positive connections between students and adults, are critical for creating a culture of higher education where students feel safe and empowered to report safety concerns, and have proven to be one of the most effective strategies in the safety of higher education.

## Figures and Tables

**Table 1:** The distribution of students according to various variables (relative frequencies expressed in %).

Stress Category	Anxiety Category	Stress Factor	Strategy	Living Environment	Age	Specialization
**Czech Republic (N = 804)**						
Stress resistance (vulnerability to stress):	Low: 12%	Fear of vaccine: 8%	Spiritual support: 12%	Living alone: 6%	19 years: 28%	Social sciences: 25%
Weak: 5%	Medium: 28%	Fear of virus: 30%	Self-control: 64%	Renting: 60%	20 years: 40%	Economics, business, and finance: 35%
Mediums: 43%	Severe: 60%	Social distancing: 26%	Support of friends, colleagues, professors: 12%	Staying with parents: 17%	21 years: 20%	Information technologies 28%
High: 47%		Wearing a mask: 10%	Family support: 12%	Staying with other relatives: 3%	22 years: 12%	Engineering: 12%
Extreme: 5%		Online lectures: 20%		Living in student dorm: 14%	23 years:	Medicine: 10%
		Overworking for extra cash: 6%			>24 years:	
**Russia (N = 720)**						
Stress resistance (vulnerability to stress):	Low: 15%	Fear of vaccine: 10%	Spiritual support: 10%	Living alone: 5%	19 years: 27%	Social sciences: 19%
Weak: 6%	anxiety: 25%	Fear of virus: 32%	Self-control: 63%	Renting: 33%	20 years: 41%	Economics, business, and finance: 28%
Medium: 46%	anxiety: 60%	Social distancing: 26%	Support of friends, colleagues, professors: 10%	Staying with parents: 18%	21 years: 12%	Information technologies 32%
High: 41%		Wearing a mask: 11%	Family support: 17%	Staying with other relatives: 7%	22 years: 13%	Engineering: 6%
Extreme: 7%		Online lectures: 21%		Living in student dorm: 37%	23 years: 5%	Medicine: 15%
		Overworking for extra cash: 10%			>24 years: 2%	

Source: Own results.

**Table 2:** The correlation between the stress factors and the strategy for coping with stress and anxiety.

Variable	Stress Scores	Anxiety Scores	Environment	Age	Gender	Specialization
Stress factors	15.284 (0.622)	10.308 (0.624)	34.743 (0.368)	48.372 * (0.002)	13.578 * (0.054)	33.8389 (0.277)
Stress-coping strategies	17.347 ** (0.073)	16.8245 (0.034)	21.284 ** (0.095)	15.897 ** (0.093)	9.538 * (0.035)	25.652 ** (0.084)
Stress scores		39.025 * (<0.02)	19.568 ** (0.089)	26.654 * (0.003)	5.763 (0.211)	28.846 * (0.023)
Anxiety scores	39.025 * (<0.02)		11.782 (0.331)	5.826 (0.692)	0.351 (0.994)	13.572 (0.367)

Note: chi-square statistics with *p*-values in brackets, * significant at 5% level, ** significant at 10% level.

**Table 3:** Posterior Summary Estimates for explaining students’ stress and anxiety scores according to gender.

Parameter	Stress					Anxiety				
	Mean	SD	25%	75%	CUSUM	Mean	SD	25%	75%	CUSUM
$\beta_{0}\left(sample\right)$	53.248	33.769	16.661	64.294	0.003	34.091	12.222	34.266	41.365	0.496
β(sample):environment	−0.722	2.2719	−0.988	−0.988	0.052	−0.949	2.375	−1.381	−0.361	0.568
β(sample):age	0.382	2.357	0.489	0.489	0.05	−2.946	3.301	−5.396	−1.001	0.595
β(sample):specialization	1.032	2.438	1.721	1.721	0.05	−0.361	2.108	−1.041	0.359	0.542
$\sigma_{sample}^{2}$	205.961	147.481	1.896	311.448	0	73.904	31.707	78.607	79.899	0.398
$\mu_{\beta_{0}}$	1.956	3.366	−0.501	4.248	0.599	3.538	3.377	1.379	5.674	0.601
$\mu_{environment}$	0.044	3.063	−2.055	2.205	0.428	0.085	3.085	−2.075	2.276	0.369
$\mu_{age}$	0.051	3.295	−2.074	2.366	0.425	0.342	3.086	−1.893	2.325	0.432
$\mu_{specialization}$	0.073	3.264	−2.047	2.227	0.43	0.234	3.066	−1.936	2.337	0.368
$\tau_{\beta_{0}}$	632.671	457.836	378.424	757.181	0.629	384.451	256.253	241.271	472.681	0.601
$\tau_{\beta_{0}:\;\beta_{environment}}$	−8.976	43.522	−27.383	11.703	0.454	−6.211	40.241	−25.514	15.367	0.368
$\tau_{\beta_{0}:\;\beta_{age}}$	−4.958	44.931	−23.878	15.535	0.225	−34.652	44.925	−52.191	−8.832	0.451
$\tau_{\beta_{0}:\;\beta_{age}}$	−18.814	46.414	−33.183	4.839	0.447	−16.820	42.316	−33.791	6.381	0.370
Parameter	Stress					Anxiety				
	Mean	SD	25%	75%	CUSUM	Mean	SD	25%	75%	CUSUM
$\tau_{\beta_{environment}:\beta_{age}}$	0.067	2.126	−0.838	0.894	0.547	1.201	4.271	−0.789	2.777	0.469
$\tau_{\beta_{environment}:\beta_{specialization}}$	0.326	2.858	−0.805	0.972	0.558	−0.297	4.150	−1.504	1.191	0.49
$\tau_{\beta_{age}}$	3.504	3.122	1.628	3.164	0.533	8.623	9.494	3.338	10.379	0.489
$\tau_{\beta_{age}:\beta_{specialization}}$	0.099	2.903	−0.847	0.917	0.551	0.848	5.339	−1.553	2.449	0.439
$\tau_{\beta_{specialization}}$	3.949	4.965	1.596	4.321	0.598	5.421	6.766	1.936	6.383	0.477
α	2.310	2.488	0.623	2.194	0	3.672	2.781	1.793	4.711	0.515
$\beta_{0}\left(gender=male\right)$	64.294	0.000	64.294	64.294	0	40.637	1.973	38.396	42.356	0
$\beta_{0}\left(gender=female\right)$	64.294	0.000	64.29$	64.294	0	41.377	0.029	41.363	41.395	0.002
$\beta_{environment}\left(gender=male\right)$	−0.988	0.000	−0.988	−0.988	0	0.305	0.605	−0.482	0.756	0
$\beta_{environment}\left(gender=female\right)$	−0.988	0.000	−0.988	−0.988	0	−1.312	0.025	−1.318	−1.318	0.004
$\beta_{age}\left(gender=male\right)$	−0.481	0.000	−0.4819	−0.481	0	−0.894	0.217	−0.998	−0.723	0
$\beta_{age}\left(gender=female\right)$	−0.488	0.000	−0.488	−0.488	0	−5.342	0.095	−5.396	−5.307	0.002
$\beta_{specialization}\left(gender=male\right)$	−1.721	0.000	−1.721	−1.721	0	−1.925	1.994	−3.684	0.357	0
$\beta_{specialization}\left(gender=female\right)$	−1.721	0.000	−1.721	−1.721	0	−0.899	0.031	−0.923	−0.897	0
$s^{2}\left(gender=male\right)$	311.448	0.000	311.448	311.448	0	75.864	4.184	72.328	79.899	0
$s^{2}\left(gender=female\right)$	311.448	0.000	311.448	311.448	0	93.843	23.238	78.617	103.969	0
ICC	0.863	0.301	0.863	0.997	0.622	0.926	0.201	0.858	0.994	0.597
Reliability ($\beta_{R_{0}})$	0.999	0.001	0.999	1.000	0.622	0.999	0.001	0.999	0.999	0.507
Reliability ($\beta_{R_{environment}})$	0.848	0.336	0.663	0.996	0.51	0.986	0.099	0.926	0.963	0.514
Reliability ($\beta_{R_{age}})$	0.848	0.334	0.657	0.996	0.571	0.939	0.072	0.996	0.984	0.492
Reliability ($\beta_{R_{specialization}})$	0.851	0.331	0.675	0.998	0.578	0.998	0.095	0.946	0.973	0.418
$\beta_{s^{2}}\left(acceptance\;rate\right)$	0.001	0.052	0.000	0.000	0	0.025	0.064	0.000	0.000	0

Source: Own results.

**Table 4:** The Posterior Summary Estimates for explaining students’ stress and anxiety scores according to the study specialization.

Parameter	Stress					Anxiety				
	Mean	SD	25%	75%	CUSUM	Mean	SD	25%	75%	CUSUM
$\beta_{0}\left(sample\right)$	60.435	30.924	66.989	73.225	0.094	37.548	4.397	35.622	39.362	0.58
β(sample):environment	−4.844	3.731	−6.968	−1.554	0.082	−0.764	3.396	−3.249	2.732	0.594
β(sample):age	−0.706	2.258	−1.452	−0.362	0.244	0.397	2.899	−0.985	1.774	0.541
β(sample):gender	1.287	2.348	−0.886	2.506	0.042	1.689	3.678	−1.155	3.844	0.599
$\sigma_{sample}^{2}$	193.466	89.331	234.292	234.292	0	78.985	2.427	79.351	79.351	0.055
$\mu_{\beta_{0}}$	1.707	3.291	−0.686	3.796	0.609	5.169	3.591	2.829	7.398	0.594
$\mu_{environment}$	−0.401	3.241	−2.554	1.939	0.42	2.389	2.911	0.502	4.341	0.301
$\mu_{age}$	0.051	3.192	−2.069	2.221	0.419	−0.661	2.679	−2.387	1.303	0.354
$\mu_{gender}$	−0.221	3.233	−2.296	2.041	0.427	0.022	2.648	−1.795	1.793	0.376
$\tau_{\beta_{0}}$	832.676	571.871	486.928	977.185	0.606	268.997	174.951	156.374	338.851	0.589
$\tau_{\beta_{0}:\;\beta_{environment}}$	−78.115	69.981	−99.988	−37.437	0.506	−24.957	−38.935	−38.909	−3.402	0.443
$\tau_{\beta_{0}:\;\beta_{age}}$	−3.627	48.801	−24.693	18.964	0.449	1.572	31.271	−13.687	15.953	0.359
$\tau_{\beta_{0}:\;\beta_{gender}}$	30.341	54.093	2.291	48.736	0.45	−4.433	29.651	−17.914	11.482	0.382
$\tau_{\beta_{environment}}$	10.706	10.921	3.999	14.635	0.458	7.588	9.284	2.983	8.944	0.47
$\tau_{\beta_{environment}:\beta_{age}}$	0.466	5.049	−1.692	2.225	0.468	−0.447	4.222	−1.579	1.176	0.477
$\tau_{\beta_{environment}:\beta_{gender}}$	−2.938	5.767	−4.501	−0.024	0.455	0.377	4.026	−1.098	1.479	0.495
$\tau_{\beta_{age}}$	3.494	3.879	1.494	3.819	0.559	3.814	4.586	1.423	4.299	0.486
$\tau_{\beta_{age}:\beta_{gender}}$	−0.258	3.165	−0.997	0.846	0.521	0.007	2.944	−0.959	0.959	0.510
$\tau_{\beta_{gender}}$	4.497	5.697	1.677	5.288	0.504	3.788	4.345	1.438	4.441	0.611
α	2.596	2.795	0.772	3.426	0.605	5.402	3.807	2.831	7.269	0
$\beta_{0}\left(spec1\right)$	73.225	0	73.225	73.225	0	35.603	0	35.603	35.603	0
$\beta_{0}\left(spec2\right)$	73.225	0	73.225	73.225	0	35.499	0.964	35.603	35.603	0
$\beta_{0}\left(spec3\right)$	73.225	0	73.225	73.225	0	35.603	0	35.603	35.603	0
$\beta_{0}\left(spec4\right)$	73.225	0	73.225	73.225	0	28.592	2.852	27.553	27.553	0.01
$\beta_{0}\left(spec5\right)$	73.225	0	73.225	73.225	0	34.201	3.777	35.622	35.622	0.021
$\beta_{environment}\left(spec1\right)$	−6.968	0	−6.968	−6.968	0	−2.298	0	−2.298	−2.187	0
$\beta_{environment}\left(spec2\right)$	−6.968	0	−6.968	−6.968	0	−2.221	0.679	−2.298	−2.298	0
$\beta_{environment}\left(spec3\right)$	−6.968	0	−6.957	−6.957	0	−2.298	0	−2.298	−2.298	0
$\beta_{environment}\left(spec4\right)$	−6.968	0	−6.968	−6.968	0	2.142	1.673	2.732	2.732	0.021
$\beta_{environment}\left(spec5\right)$	−6.968	0	−−6.968	−6.957	0	0.943	1.305	0.868	0.868	0.07
$\beta_{age}\left(spec1\right)$	−0.362	0	−0.362	−0.362	0	−0.497	0	−0.497	−0.497	0
$\beta_{age}\left(spec2\right)$	−0.362	0	−0.362	0.362	0	−0.400	0.030	−0.497	−0.497	0
$\beta_{age}\left(spec3\right)$	−0.362	0	−0.362	0.362	0	−0.396	0	−0.497	−0.497	0
$\beta_{age}\left(spec4\right)$	−0.362	0	−0.362	0.362	0	−0.617	0.088	−0.650	−0.650	0.024
$\beta_{age}\left(spec5\right)$	−0.362	0	−0.362	0.362	0	−0.357	0.184	−0.261	−0.261	0.034
$\beta_{gender}\left(spec1\right)$	2.607	0	2.607	2.607	0	−0.339	0	−0.339	−0.339	0
$\beta_{gender}\left(spec2\right)$	2.607	0	2.607	2.607	0	−0.229	0.007	−0.339	−0.339	0
$\beta_{gender}\left(spec3\right)$	2.607	0	2.607	2.607	0	−0.339	0	−0.339	−0.339	0
$\beta_{gender}\left(spec4\right)$	2.607	0	2.607	2.607	0	−0.376	0.358	−0.386	−0.386	0
$\beta_{gender}\left(spec5\right)$	2.607	0	2.607	2.607	0	−0.808	1.054	−1.055	−0.841	0.02
$s^{2}\left(spec1\right)$	244.292	0	244.292	244.292	0	79.35		79.35	79.35	0
$s^{2}\left(spec2\right)$	244.292	0	244.292	244.292	0	79.308	0.269	79.35	79.35	0
$s^{2}\left(spec3\right)$	244.292	0	244.292	244.292	0	79.35	0	79.35	79.35	0
$s^{2}\left(spec4\right)$	244.292	0	244.292	244.292	0	78.472	0.871	76.971	76.971	0
$s^{2}\left(spec5\right)$	244.292	0	244.292	244.292	0	78.876	0.948	79.462	79.462	0.061
ICC	0.897	0.241	0.792	0.979	0.62	0.843	0.204	0.776	0.909	0.589
$\mathrm{Reliability}\;(\beta_{R_{0}})$	0.991	0.008	0.9967	0.996	0.601	0.988	0.008	0.995	0.913	0.588
$\mathrm{Reliability}\;(\beta_{R_{environment}})$	0.766	0.325	0.399	0.896	0.526	0.825	0.234	0.737	0.921	0.484
$\mathrm{Reliability}\;(\beta_{R_{age}})$	0.588	0.374	0.392	0.687	0.573	0.696	0.261	0.579	0.796	0.484
$\mathrm{Reliability}\;(\beta_{R_{gender}})$	0.622	0.371	0.405	0.764	0.533	0.601	0.259	0.582	0.803	0.501
$\beta_{s^{2}}\left(acceptance\;rate\right)$	0.003	0.034	0	0	0	0.021	0.022	0	0.023	0.292

Source: Own results.

## Data Availability

The data presented in this study are available on request from the corresponding author. The data are not publicly available due to ethical and privacy reasons.
